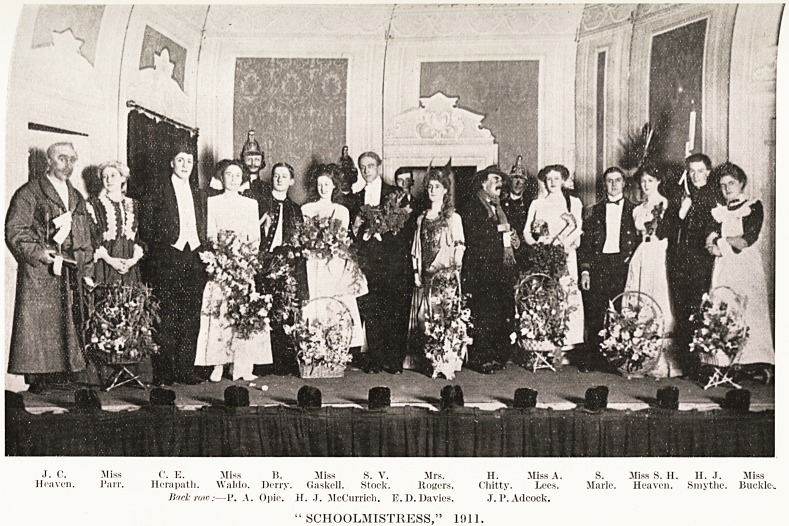# The History of the Bristol Medical Dramatic Club

**Published:** 1936

**Authors:** A. L. Flemming


### THE HISTORY OF THE BRISTOL MEDICAL DRAMATIC CLUB.

BY

A. L. Flemming, M.B., Ch.B.

---

The origin of this Club dates back to 1880, when a group of medical students and their friends gave a performance in the Alexandra Hall, Clifton, in aid of the Royal Infirmary and the General Hospital. The venture was so successful that it was not long before the annual performances took their place in the regular medical and social life of the district. The original members were H. W. Husbands, H. Simmons, L. E. A. Salmon, F. J. Wethered and W. Lacroix. Their efforts were well received on all hands, and won for them the support of their professional colleagues and of an enthusiastic and generous public. They were fortunate in having the valuable assistance of certain members of the acting profession whose services were kindly lent by Mr. Chute, of Prince's Theatre. Among the latter were Miss Ethel Vane, Mr. Stephenson and Mr. Arnold, who helped to produce some of the early plays.

There can be no doubt that the phenomenal way in which the Club won popularity from its very inception was due to good acting backed up by the efficient organization of Husbands, Simmons, Watson-Williams and Alec Spencer.

Having been launched under such exceptionally favourable conditions and with a host of influential friends, the venture went forward on its successful career, producing one, and occasionally two, plays every year. The first break was in 1887, but in 1888 Mr. Lace and Mr. Dundas Edwards put on "The Heir at Law." There was no performance during the Great War, nor from 1932 to 1934. With the exception of 1885, when "Dearest Emma" was played at the private residence of Mrs. Colthurst, in Clifton, all performances took place at the Alexandra Hall until that building was closed in 1897. After this the All Saints Hall was used till 1924, from when till 1931 accommodation was provided in the Victoria Rooms. The last two performances have taken place in the Clifton Parish Hall.

In 1899 a resolution was passed "that in future all seats should be charged for," and Mrs. Cross was asked to organize a ladies' committee for the sale of tickets. This meant the end of the old system of collecting money in the room, a method which had often given good results, as in 1882 and 1895, when £112 and £101 were taken.

It has been the practice to divide any profits which may accrue between the General Hospital and the Infirmary, but in 1889 they were devoted to the Building Fund for the new Medical School.*

A feature of the organization has, throughout, been the attention to details given by the Committee, whose energies can best be appreciated by a perusal of the minute books. For many years the Club's fortunes were jealously watched over by such

* The other hospitals to which proceeds have been allotted were the Orthopædic Hospital in 1912 and the Bristol Eye Hospital in 1913. From time to time small sums have been devoted to special charities, such as the Ottawa Fire Relief Fund in 1900 and the Soldiers' and Sailors' Relief Fund in 1920.

PLATE XXVII

*Names from left to right :*—J. A. Nixon, L. A. Moore, A. L. Sheppard, Fred Emery, L. Shingleton Smith, A. E. Iles, G. Munro Smith, H. R. B. Hull, S. V. Stock, C. H. Hart, E. H. E. Stack, S. P. Eyre, J. C. Heaven.

Mrs. F. W. Rogers, Miss Ida Prichard, Miss M. Parr, Miss Irene Prichard.

"THE PICKPOCKET," 1906.

### PLATE XXVIII

H. W. Husbands.
↓

↑ Miss Marie Collins. ↑ W. L. Lacroix.

"ICI ON PARLE FRANÇAIS," 1880.

George. The Father, Mr. Westlake. William Burridge.

THREE CHARACTERS IN "BARNET'S FOLLY," 1936.

enthusiastic champions as Mr. Munro Smith, Dr. Davies, Dr. Heaven, Dr. Stack, Mr. Lansdown, Mr. Stock and Dr. L. A. Moore, and they are now the care of Dr. Hastings Moore.

A large share of the credit for the long and successful career of the Club is due to the many gifted ladies who have taken part in the acting or played in the orchestra.

On a few occasions the musical arrangements were in the hands of non-medical friends, such as Miss Fyffe, Mr. Frank Gardiner, Mr. Bucknall and Mr. Harold Bernard, but, with these exceptions, the conductor of the orchestra has always been a member of the profession, as: Little, Meaden, McEnnery, Bullen, Nixon, Penglaze, Heaven and Claremont.

The success of this organization, after such a vigorous start at the hands of its originators in 1880, reminds one of the saying: "The future destiny of the child is always the work of the mother."

The list of plays produced during the fifty-seven years that the Club has been in existence is too long to be reviewed in detail in these pages, but results have proved the wisdom of the selectors in keeping almost entirely to comedy. The encouraging reception afforded to the first comedy attempted, Byron's "Weak Woman," led to a second performance later in the same year, when the Company acted Byron's "£100,000," by which they established their popularity with the public and amongst their colleagues.

They were fortunate in having the assistance of Miss Ethel Vane, Miss Bramhall, and Miss Barnes, and in being coached and managed by Stephenson and Arnold from the New Theatre. The students who acted on this occasion were Husbands, Simmons, Spencer, Lacroix, and Capron, and much of the organization was carried out by their fellow-student, P. Watson-Williams.

In the following season the cast was strengthened by the inclusion of Mr. Munro Smith in the rôle of Jacob Mortmain in "Black Sheep." Another source of strength on this occasion was Mr. C. Matthews, who took the part of Toby Tweedleton in "Tweedleton's Tail Coat." Good fortune again favoured the Club during the next season, when it had the advantage of Dr. Weatherly's artistic impersonation of Cassidy, the manservant in "Old Soldiers." The performances given annually had gained considerable popularity, when in 1886 an experienced and talented cast gave a successful rendering of "Old Sailors," Mr. Munro Smith, Mr. Matthews, Husbands, Miss Barnes and Miss Bramhall winning fresh laurels for themselves and renewed kudos for the Club. Such successful performances as those of "London Assurance," "Dandy Dick," and "Our Boys" maintained the high reputation of the medical dramatics; Pinero's "The Magistrate" and "The School Mistress" gave the actors and producers a chance of displaying their skill, but "Held by the Enemy," with its large cast, proved to be of special popularity among the actors.

The Club has never been without an experienced producer: it enjoyed twenty years of help in this capacity from Dr. Davies and Dr. Heaven, and subsequently the function has been undertaken by Dr. Stock and Mr. Jackman till last year, when the reins passed to the able hands of Dr. Hastings Moore. As to the actors, we cannot think of the past performances without being reminded of Mr. Munro Smith, Mr. Lansdown, Dr. Davies, Dr. Heaven, Mr. Stack or Mr. Bernard Cridland.

F. J. Wethered. L. E. A. Salmon.

H. Simmons. W. L. Lacroix. H. W. Husbands. Miss Marie Collins

"£100,000."

PLATE XXIX

PLATE XXX

R. G. P. Lansdown.

"DANDY DICK," 1899.

Mr. Munro Smith was a natural artist whose sense of humour and genial power made him a great favourite with audience and fellow-actors alike. His character sketches as Gregory Grumbledon the would-be invalid in "The Pickpocket," as Perkyn Middlewick the illiterate butterman in "Our Boys," and as William Dott the butler in "Home, Sweet Home," were instances of his capacity for sustaining a rôle without exaggeration but with unfailing humour. Mr. Munro Smith's individuality stood as a great asset to the Club throughout his thirty-two years of membership.

Mr. R. G. P. Lansdown was a keen supporter, taking considerable pains in Committee, he was President from 1898 till 1903, and subsequently served as an energetic member of the Committee till 1914. On the occasions when he took part in the performances his careful study of action and voice brought a sense of reality to his impersonations; he was equally good, for instance, in the title rôle in "The Magistrate," as the Very Rev. Augustin Jedd the Dean in "Dandy Dick," and as Pete the farm labourer in "The Professor's Love Story," although the parts were so different. We reproduce Mr. R. C. Carter's excellent caricature of his impersonation of "The Dean."

Mr. A. Bernard Cridland was an actor of exceptional force and talent. He was a great favourite as juvenile lead. Among his successful rôles may be mentioned those of Edward Trentham the young country gentleman in "Courtship," and of Cis Farringdon in "The Magistrate." His rendering of these parts and of the others he took during the short time he remained in Bristol showed him to be an elegant artist with a keen appreciation of dramatic effect; his withdrawal from the district to practise elsewhere was a real loss to the Club.

Dr. Davies and Dr. Heaven, who were closely associated in their professional posts, worked hand in hand in their many efforts on behalf of the "Dramatics," in fact we are tempted to refer to them in one paragraph; between them they produced two plays in the year 1895, "London Assurance" in February and "Courtship" in December. In the former Dr. Heaven appeared in the rôle of Max Harkaway the country squire, and Dr. Davies was the producer: in the latter Dr. Heaven produced while Dr. Davies functioned as treasurer. This was typical of them, always ready to fill any vacancy where their services seemed called for: Dr. Heaven, for instance, when not required elsewhere, played the 'cello in the orchestra. In one capacity or another they continuously supported the Club from 1895 till 1921, and their help was of great value owing to their exceptional knowledge of all the mysteries appertaining to the theatrical stage. Dr. Heaven was always strongly opposed to the system of obtaining money from an audience by means of a collection instead of by charging for admission.

Dr. Stack came on to the Committee in 1900 and served till 1921. He was energetic in forwarding any movement which in his conception was to the advantage of the students, and soon became an ardent supporter of the "Dramatics," taking part in the acting on many occasions and often exercising his ingenuity in stage carpentry and other mechanical processes in the arranging of the stage. Among the parts he acted with great care and attention to detail were those of Mr. Walter Johnson in "The Pickpocket," and Major-General Stamburg in "Held by the Enemy." In all the characters he represented he was very careful in his study of detail in both action and

J. C. Heaven. Miss Parr. C. E. Herapath. Miss Waldo. B. Derry. Miss Gaskell. S. V. Stock. Mrs. Rogers. H. Chitty. Miss A. Lees. S. Marle. Miss S. H. Heaven. H. J. Smythe. Miss Buckle.

*Back row:*—P. A. Opie. H. J. McCurrich. E. D. Davies. J. P. Adcock.

"SCHOOLMISTRESS," 1911.

PLATE XXXI

diction. Dr. Stack was so energetic that it was difficult to know whether his chief interest lay in the actual playing or in the general organizing, but he undoubtedly helped to "make the performance go."

We cannot fail to recall the memory of the charming Dr. James Taylor, who so regularly played the double bass by the side of his old friend Mr. Eyles.

With reference to the photographs, we wish to explain that the picture of three characters in this year's play, "Barnet's Folly," is intended to illustrate the advance that has been made in the art of making-up during the past fifty years. The negative has been lent to us on condition that the identity of the actors shall not be published.

### PLAYS PERFORMED.

1880. Ici on parle Français.
1881 (Feb.). Weak Woman.
Lend me 5s.
1881 (Dec.). £100,000.
My Turn Next.
1882. Tweedleton's Tail Coat.
1882. Black Sheep.
1883. Old Soldiers.
1884. Meg's Diversion.
1884. Turn Him Out.
1885. Dearest Emma.
1886. Old Sailors.
1886. Sweethearts.
1887. (No play.)
1888. Heir at Law.
1889. Road to Ruin.
1890. Cyril's Success.
1891. Uncle.
1891. Who's Who.
1893. The Old Story.
1893. Barbara.
1894. Not such a Fool as he looks.
1895 (Feb.). London Assurance
1895 (Dec.). Courtship, or The Three Caskets.
1896. Prude's Progress.
1898. The Magistrate.
1899. Dandy Dick.
1900. The Professor's Love Story.
1901. The Man in the Street.
1901. The Parvenu.
1903. Our Boys.
1904. The Lancers.
1905. A Brace of Partridges.
1906. The Pickpocket.
1907. Jacques the Spy.
1907. Home, Sweet Home.
1908. Held by the Enemy.
1909. Jedbury Junior.
1910. An American Citizen.

1911. The School Mistress.
1912. Dr. Wake's Patient.
1913. The Manœuvres of Jane.
1914. Upper Crust.
1920. The Younger Generation.
1921. Mice and Men.
1922. Duke of Killicrankie.
1923. Tilly of Bloomsbury.
1924. The Cheerful Knave.
1925. Billeted.
1926. The Dover Road.
1927. Nothing but the Truth.
1928. The Creaking Chair.
1929. Ambrose Applejohn's Adventure.
1930. The Fourth Wall.
1931. French Leave.
1935. The High Road.
1936. Barnet's Folly.

## Figures and Tables

**Figure f1:**
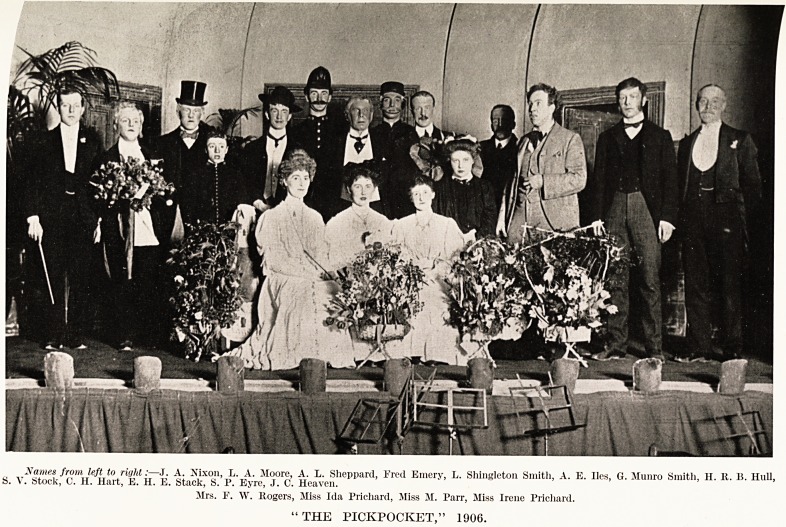


**Figure f2:**
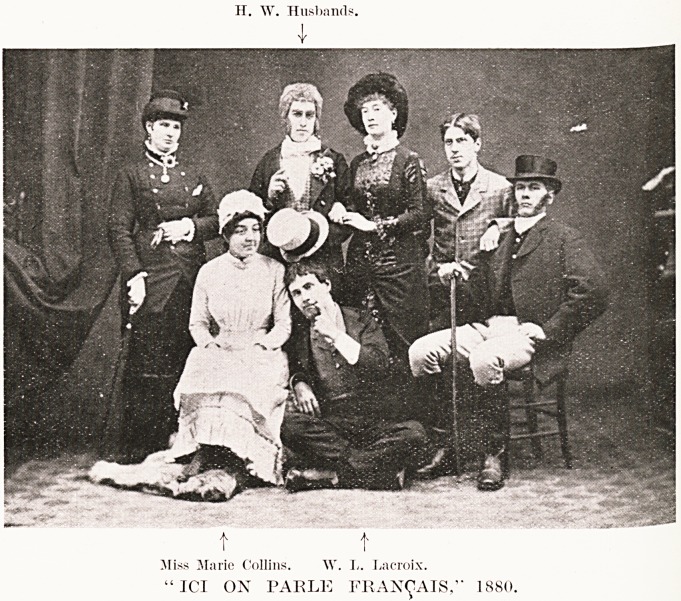


**Figure f3:**
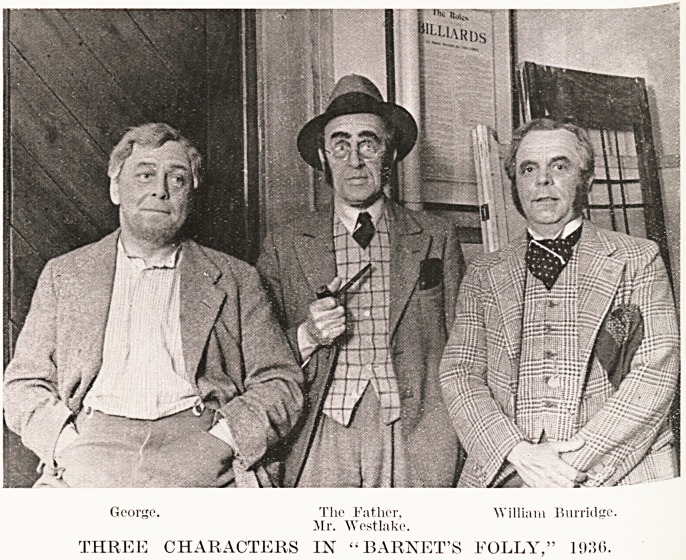


**Figure f4:**
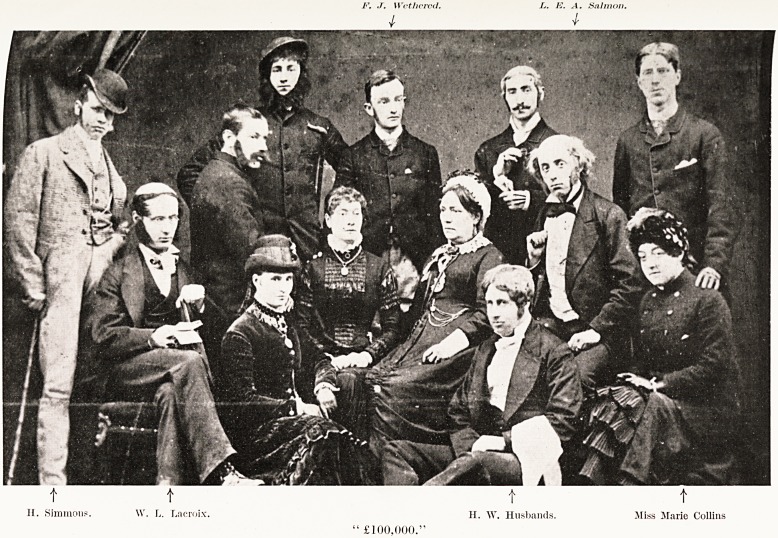


**Figure f5:**
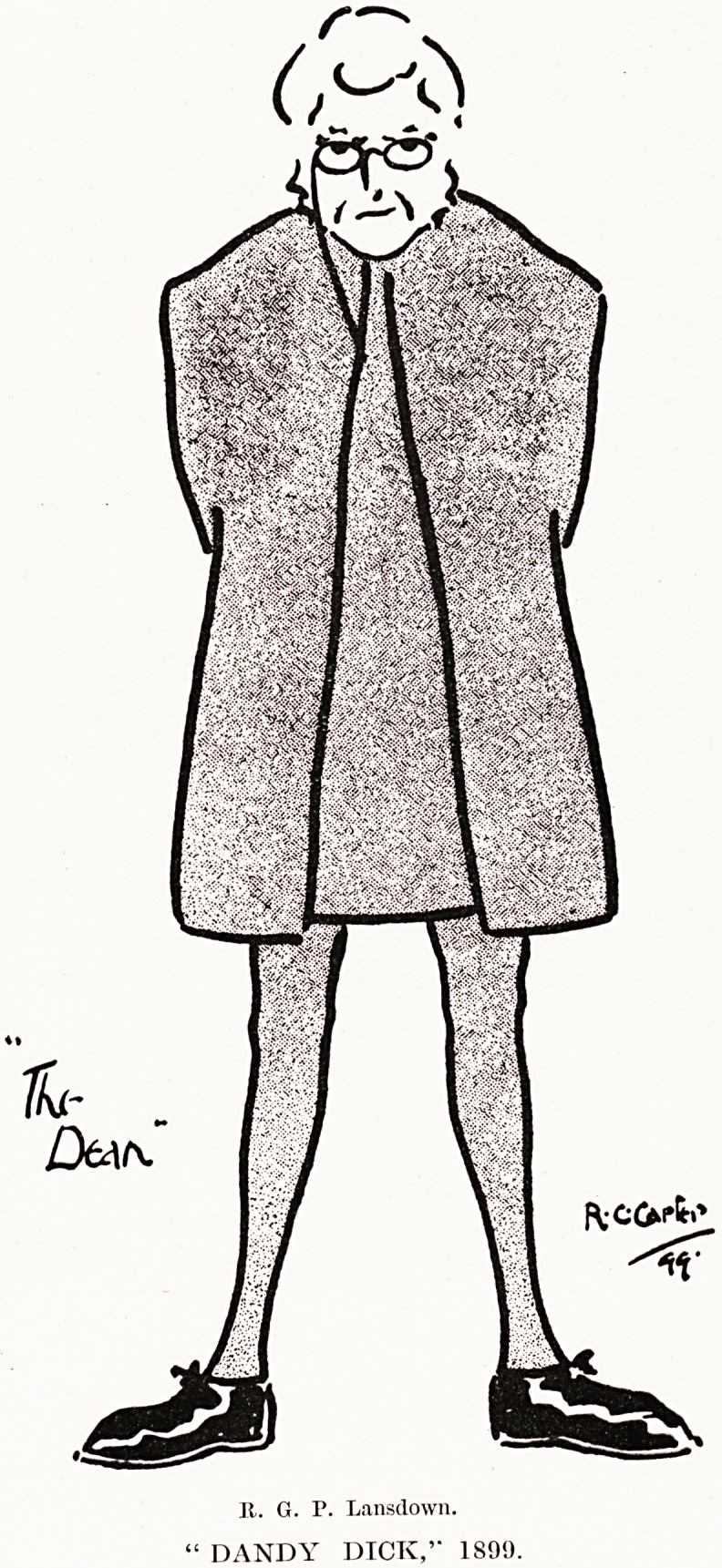


**Figure f6:**